# Investigating Disparities Related to Insurance Status and Access to Locoregional Therapies for Patients with Hepatocellular Carcinoma Awaiting Liver Transplantation

**DOI:** 10.1007/s10620-025-09462-5

**Published:** 2025-11-01

**Authors:** Soobin S. Lee, Lindsay M. Thornton, Patricia D. Jones, David S. Goldberg

**Affiliations:** 1 University of Miami Miller School of Medicine, Miami, FL 1500 NW 12thAve #1101 USA; 2 Department of Radiology, University of Miami Miller School of Medicine, Miami, FL USA; 3 Division of Digestive Health and Liver Diseases, University of Miami Health System, University of Miami Miller School of Medicine, Miami, FL USA; 4 Division of Digestive Health and Liver Diseases, University of Miami Miller School of Medicine, Miami, FL USA

**Keywords:** Liver transplantation, Waitlist dropout, MELD exceptions, Public insurance

## Abstract

**Purpose:**

Patients with hepatocellular carcinoma (HCC) and Medicaid insurance on the liver transplantation (LT) waitlist face higher risks of waitlist dropout, though mechanisms behind this disparity remain unclear. We aimed to assess differences in the use of locoregional therapy (LRT) based on insurance status and whether these differences contribute to waitlist disparities.

**Methods:**

We conducted a retrospective cohort study using Organ Procurement and Transplantation Network/United Network for Organ Sharing (OPTN/UNOS) data on adult patients (≥ 18 years) waitlisted with standardized HCC Model for End-stage Liver Disease (MELD) exceptions from 1/1/2015 to 12/31/2022. Mixed effects multiple variable logistic regression models were used to evaluate the association between insurance status and LRT receipt, adjusting for key clinical and HCC-related variables.

**Results:**

Patients with Medicaid had higher odds (OR: 1.09; 95% CI: 1.01, 1.18) of receiving LRT compared to patients with private insurance. Additionally, when comparing waitlist time following HCC MELD exception approval for our cohort, Medicaid patients experienced longer median waitlist time (206 days; IQR 88–371) compared to privately insured patients (182 days; IQR 69–313) (*p* < 0.001).

**Conclusion:**

Contrary to expectations, Medicaid patients were more likely to receive LRT than those with private insurance. These findings highlight the importance of further investigating contributing factors that facilitate these outcomes.

**Supplementary Information:**

The online version contains supplementary material available at 10.1007/s10620-025-09462-5.

## Introduction

Patients diagnosed with early- or intermediate-stage HCC have several potentially curative treatment options. For select patients, LT is associated with the longest overall survival. However, due to a shortage of donor livers and the current allocation system, patients waitlisted for HCC face waiting times of at least six months, and oftentimes more than a year [1]. As a result, locoregional treatments often serve as the bridge to LT to prevent disease progression, especially in patients facing prolonged waiting times [2].

Despite advances in LRT and increases in organ donation, not all patients waitlisted for HCC will survive to transplantation. Patients waiting for LT face risks of tumor progression and/or hepatic decompensation that result in them to be too sick to transplant. Unfortunately, there are documented disparities in waitlist survival for patients with HCC. For example, a 2019 study from Kaiser Permanente Northern California demonstrated that patients with public insurance had worse waitlist outcomes and an increased risk of waitlist dropout compared to those with private insurance [3]. However, the study did not explore the mechanism leading to this increased waitlist dropout. Given that the relationship between insurance status and waitlist outcomes is already well-established in literature, we sought to focus on a more specific relationship between insurance status and LRT.

We sought to leverage national transplant registry data to explore factors leading to disparities in waitlist survival for patients with HCC and Medicaid insurance, with a primary focus on differences in receipt of LRT. Of important consideration, our analysis focuses on a highly selected subset of Medicaid patients who have already overcome significant barriers to achieve waitlist placement.

## Methods

This retrospective cohort study used data from the Organ Procurement and Transplantation Network (OPTN)/United Network for Organ Sharing (UNOS), including the waitlist and the exception databases in the Standard Transplantation Analysis and Research (STAR) file. We included all adult patients, ≥ 18 years of age, waitlisted with an approved standardized HCC MELD exception from 1/1/2015 to 12/31/2022. The dates of the listing eras were made relevant to policy changes on October 8, 2015, and May 1, 2019, to account for potential effects on LRT accessibility. Our primary objectives focused on differences in management once waitlisted, specifically regarding receipt of LRT, and secondarily, differences in severity of illness (e.g., MELD score) and tumor burden at waitlisting. The primary study outcome was receipt of LRT among patients waitlisted with HCC. Using the exception dataset, we determined whether a patient received LRT at any time point (i.e., at the initial exception application or any time thereafter). LRT was defined as: ablative therapies (e.g., radiofrequency ablation), as well as non-ablative therapies (e.g., TACE, Y-90, external beam radiation).

We chose a binary outcome of receipt of LRT, rather than time-to-event for several reasons: (1) OPTN data do not provide dates of procedures, but only whether they were performed since the last exception approval; (2) OPTN data does not capture all treatment prior to waitlist; and (3) we wanted to focus on global receipt of LRT. As a result, a competing risk model framework was not applicable. The primary exposure of interest was insurance status, defined as: private insurance, Medicare, Medicaid, VA, and others. To evaluate the association between insurance type and receipt of LRT, we fit mixed effects multiple variable logistic regression models (outcome: receipt of LRT; random effect: transplant center). Variables tested for inclusion in our models included: gender, race/ethnicity, age, listing era, and baseline disease severity (total tumor size, alpha-fetoprotein (AFP), ascites, and bilirubin values at listing).

These models evaluate the association between key exposures and the binary outcome of receipt of LRT, while considering the baseline differences in center practice. We used a backwards variable selection process. This study was considered exempt by the Institutional Review Board at the University of Miami.

## Results

Our cohort included 49,404 adults waitlisted with HCC MELD exceptions. Patients with Medicaid were significantly younger than those with other insurance types, and more likely to be non-Hispanic Black or Hispanic (Table 1). Consistent with prior studies, we found that patients with Medicaid have a higher risk of waitlist removal for dying or becoming too sick to transplant (21.5% vs 15.6% in those with private insurance) (Table 2).

**Table 1:** Clinical and demographic data based on insurance status

Baseline clinical and demographic variables*	Private *N* = 26,317	Medicaid *N* = 6,847	Medicare *N* = 13,475	VA *N* = 1,577	Other *N* = 1,188	P-value
Male gender	20,662	4,997	9,689	1,513	909	< 0.001
Race/ethnicity						< 0.001
Non-Hispanic White	17,598	3,328	8,827	1,093	713	
Non-Hispanic Black	2,064	756	1,171	245	69	
Hispanic	4,012	1,913	2,481	179	230	
Asian	2,332	830	834	23	151	
Other**	311	110	162	37	25	
Age	58 (54, 62)	57 (52, 61)	65 (60, 68)	61 (57, 65)	59 (54, 64)	< 0.001
Era of waitlisting						< 0.001
1/1/15–10/7/15	17,280	3,947	6,548	825	719	
10/8/15–4/30/19	5,038	1,611	3,486	435	152	
5/1/19–12/31/22	3,999	1,289	3,441	317	317	
Serum bilirubin	1.3 (0.8, 2.2)	1.4 (0.9, 2.3)	1.2 (0.8, 2.0)	1.3 (0.8, 2.1)	1.4 (0.8, 2.2)	< 0.001
Ascites						< 0.001
Absent	13,374	3,098	6,677	835	558	
Slight	10,520	3,045	5,657	621	493	
Moderate	1,802	588	1,043	114	79	
N/A	13	3	10	0	1	
Total tumor size	2.5 (1.8, 3.6)	2.4 (1.5, 3.5)	2.4 (1.3, 3.5)	2.1 (0.0, 3.2)	2.5 (1.5, 3.7)	< 0.001
AFP	9 (4, 34)	9 (5, 33)	8 (4, 26)	7 (4, 24)	8 (4, 27)	< 0.001

Abbreviations: AFP = alpha fetoprotein

^*^Data presented as median (IQR) or N (%)

^**^Includes American Indian or Alaska Native, Native Hawaiian/other Pacific Islander, and other

**Table 2:** Waitlist outcomes in relation to insurance status

	**Waitlist outcome**			
Insurance	Died/too sick	Transplanted	Other	Total
Private	4,114 (15.6%)	18,307 (69.6%)	3,896 (14.8%)	26,317
Medicaid	1,472 (21.5%)	4,267 (62.3%)	1,108 (16.2%)	6,847
Medicare	2,720 (20.2%)	8,427 (62.5%)	2,328 (17.3%)	13,475
VA	281 (17.8%)	992 (62.9%)	304 (19.3%)	1,577
Other	149 (12.5%)	769 (64.7%)	270 (22.7%)	1,188

Abbreviations: VA = Veterans Affairs

When comparing measures of baseline disease severity among insurance status, we found that there was no meaningful difference. In other words, Medicaid patients did not present with significantly worse illness at baseline compared to those with private insurance. Overall, most patients (75.1%) in our cohort received LRT; within this group, 74.3% of Medicaid patients and 72.8% of patients with private insurance received LRT (Table 3). Those who received LRT presented with better liver function and fewer complications of portal hypertension (HTN), compared to those who did not receive LRT. Specifically, those who received LRT presented with significantly lower values in baseline serum bilirubin, total tumor size, and AFP (Table 3).

**Table 3:** Patient demographics and clinical factors in relation to receipt of LRT

Baseline clinical and demographic variables	No LRT *N* = 12,327	LRT *N* = 37,077	P-value
Male gender	9,305	28,465	0.004
Insurance			< 0.001
Private	7,156	19,161	
Medicaid	1,763	5,084	
Medicare	2,773	10,702	
VA	260	1,317	
Other	375	813	
Race/ethnicity			< 0.001
Non-Hispanic White	8,094	23,375	
Non-Hispanic Black	1,065	3,240	
Hispanic	2,047	6,768	
Asian	986	3,184	
Other**	135	510	
Age	57 (52, 63)	61 (56, 65)	< 0.001
Era of waitlisting			< 0.001
1/1/2015–10/7/2015	10,068	19,251 (51.9%)	
10/8/2015–4/30/2019	1,373 (11.1%)	9,349 (25.2%)	
5/1/2019–12/31/2022	886 (7.2%)	8,477 (22.9%)	
Serum bilirubin	1.6 (1.0, 2.7)	1.2 (0.8, 2.0)	< 0.001
Ascites			< 0.001
Absent	4,524 (38.7%)	20,018 (54.3%)	
Slight	5,819 (49.8%)	14,517 (39.4%)	
Moderate	1,328 (11.4%)	2,298 (6.2%)	
N/A	8 (0.1%)	19 (0.1%)	
Total tumor size	2.6 (2.0, 3,5)	2.4 (1.2, 3.5)	< 0.001
AFP	10 (4, 49)	8 (4, 28)	< 0.001

AFP = alpha fetoprotein

VA = Veterans Affairs

^*^Data presented as median (IQR) or N (%)

^**^Includes American Indian or Alaska Native, Native Hawaiian/other Pacific Islander, and other

The final models, with primary outcome of LRT receipt, included the following variables: insurance, gender, ethnicity, age at listing, listing era, and measures of baseline disease severity (ascites, bilirubin, total tumor size, and AFP at listing). Despite adjustment for these variables, patients with Medicaid (OR: 1.09, CI: 1.01, 1.18) or VA insurance (OR: 1.50, CI: 1.28, 1.77) insurance were significantly more likely to receive LRT (Table 4).

**Table 4:** Primary outcome of LRT receipt in relation to clinical and demographic variables

Baseline clinical and demographic variables	Odds Ratio (95% CI)	P-value
Gender		
Male	Reference	
Female	0.89 (0.84, 0.95)	< 0.001
Insurance		
Private	Reference	
Medicaid	1.09 (1.01, 1.18)	0.021
Medicare	1.02 (0.95, 1.08)	0.634
VA	1.50 (1.28, 1.77)	< 0.001
Other	0.85 (0.72, 0.99)	0.041
Race/ethnicity		
Non-Hispanic White	Reference	
Non-Hispanic Black	1.09 (0.99, 1.19)	0.069
Hispanic	0.97 (0.90, 1.04)	0.341
Asian	1.06 (0.96, 1.17)	0.241
American Indian/Alaska Native	1.12 (0.81, 1.54)	0.487
Native Hawaiian/Other Pacific Islander	1.20 (0.67, 2.15)	0.540
Multiracial	1.32 (0.87, 1.99)	0.189
Age	1.03 (1.02, 1.03)	< 0.001
Era of waitlisting		
1/1/2015–10/7/2015	Reference	
10/8/2015–4/30/2019	3.33 (3.08, 3.59)	< 0.001
5/1/2019–12/31/2022	3.39 (3.12, 3.68)	< 0.001
Serum bilirubin	0.84 (0.83, 0.85)	< 0.001
Ascites		
Absent	Reference	
Slight	0.63 (0.60, 0.66)	< 0.001
Moderate	0.41 (0.38, 0.45)	< 0.001
N/A	7.99 (0.26, 248.23)	0.236
Total tumor size	0.89 (0.88, 0.91)	< 0.001
Baseline AFP	1.00 (1.00, 1.00)	< 0.001

AFP = alpha fetoprotein

VA = Veterans Affairs

**Fig. 1:**
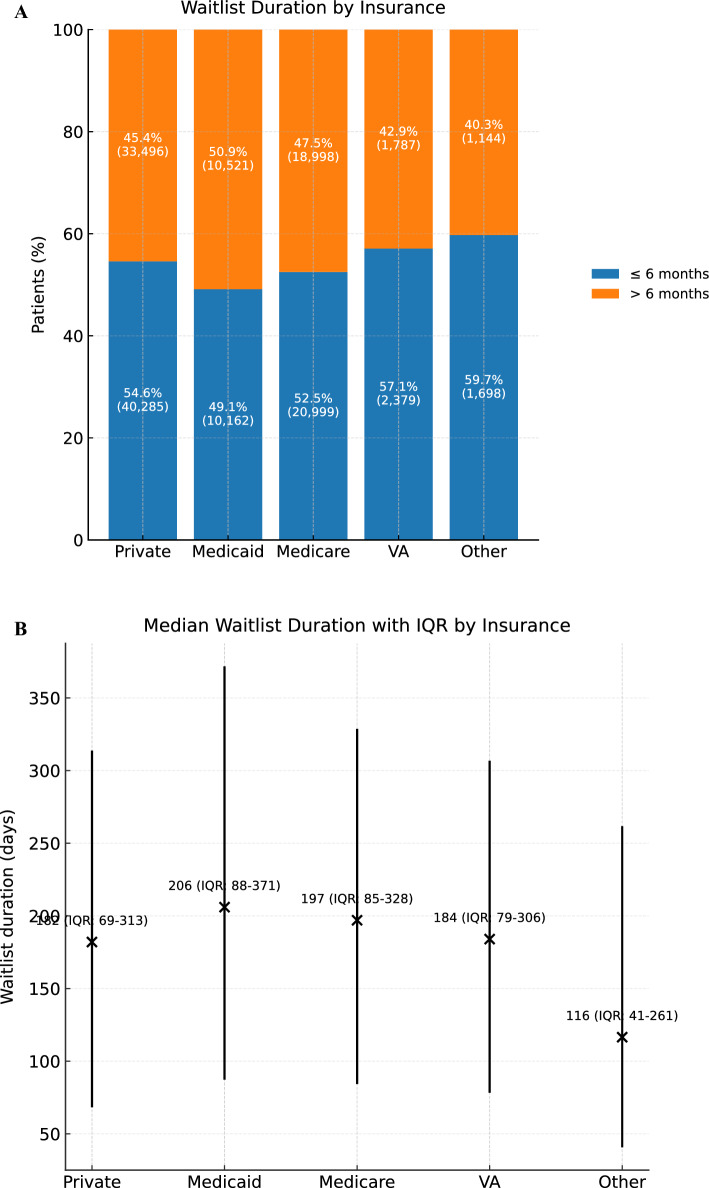
**A**: Duration on waitlist for the overall cohort stratified in relation to insurance status. **B**: Median duration on waitlist for the overall cohort stratified in relation to insurance status

In evaluating duration of waitlist, we found that Medicaid patients had the longest median waitlist time following HCC MELD exception approval (206 days [IQR 88–371]) compared to those with private insurance (182 days [IQR 69–313]) (*p* < 0.001) (Fig. 1). A greater proportion of Medicaid patients (50.9%) waited more than six months, compared to 45.4% of privately insured patients (Fig. 1).

Medicaid beneficiaries comprised a significantly higher proportion of patients with HCC exceptions waitlisted in “long-wait regions” (Regions 1, 5, and 9; 19.5% of HCC patients had Medicaid) compared to “mid-wait regions” (2, 4, 6, 7, and 8; 11.4% of HCC patients had Medicaid) and “short-wait regions” (3, 10, 11; 9.9% of HCC patients had Medicaid) (Tables 5 and 6). However, within each wait-time category, the median waiting time for Medicaid beneficiaries was longer than those with private insurance.

**Table 5:** Regional categorization of centers based on wait durations in relation to insurance status

	**Region Category**			
Insurance	Short-Wait Region	Mid-Wait Region	Long-Wait Region	Total
Private	6,256 (53.5%)	10,821 (52.6%)	9,240 (54.3%)	26,317
Medicaid	1,163 (9.9%)	2,365 (11.4%)	3,319 (19.5%)	6,847
Medicare	3,527 (30.2%)	5,906 (28.5%)	4,042 (23.8%)	13,475
VA	420 (3.6%)	1,133 (5.5%)	24 (0.1%)	1,577
Other	329 (2.8%)	478 (2.3%)	381 (2.2%)	1,188

**Table 6:** Median wait time duration of private vs Medicaid patients in relation to center region

	**Wait time duration, days**	
Region Category	Private Insurance	Medicaid Insurance
Short-Wait	252 [IQR: 129, 443]	270 [IQR: 143, 469]
Mid-Wait	176 [IQR: 71, 286]	182 [IQR: 73, 298]
Long-Wait	86 [IQR: 30, 198]	98 [IQR: 31, 214]

## Discussion

In this national study of patients waitlisted with HCC exceptions, we explored relevant factors leading to disparities in waitlist outcomes for patients with HCC and Medicaid insurance, with a primary focus on receipt of LRT as a mechanism for such disparities.

Previous studies have highlighted disparities in HCC treatment and survival outcomes based on insurance type [4]. For example, an analysis of the National Cancer Database revealed worse survival outcomes with older age, Black race, rurality, public insurance, and lower income [5]. Another 2018 study, focusing on tumor stage and likelihood to receive care, demonstrated that patients with Medicaid or those that were uninsured were less likely to have localized tumor stage at diagnosis and less likely to receive treatment [6]. These studies, along with the previously mentioned study from Kaiser Permanente Northern California, indicate toward the well-established association between insurance type and waitlist disparities among patients with HCC. Our study acknowledges this association between public insurance and worse waitlist outcomes, while further building upon this understanding by focusing on the role of LRT access in shaping these disparities.

Our main findings yielded unexpected results regarding access to LRT. Contrary to our initial hypotheses, patients with Medicaid were not less likely to receive LRT; in fact, they had higher rates of LRT utilization compared to those with private insurance. Further, our models highlight that severity of baseline disease and tumor burden are not significantly associated with differences in waitlist disparities; in other words, in our cohort, these characteristics do not play a role in explaining such waitlist disparities. This appears to challenge conventional assumptions that public insurance necessarily limits access to advanced care.

In attempting to examine the mechanism behind this finding, we considered that a longer duration of time on waitlist could contribute to greater likelihood of receiving LRT. In other words, Medicaid patients may not necessarily be receiving more LRT as a function of better access, but rather are not less likely to receive it, due to prolonged waitlist exposure. Indeed, patients with Medicaid were more likely to have longer wait times following HCC MELD exception approval. However, the slightly longer duration of 24 days does not seem to be clinically substantial and play a significant factor in our findings.

To further explore the factors underlying our findings, we evaluated whether regional variation in transplant center wait times- categorized as long-wait, mid-wait, and short-wait centers- might explain the observed differences. As anticipated, long-wait regions had a greater proportion of Medicaid patients. However, when stratified by region type, the observation of longer wait times for Medicaid patients remained consistent across all categories. This suggests that the geographic distribution of Medicaid patients does not account for our results.

Another potential explanation for our findings may lie in differences in treatment received prior to listing- information that is not comprehensively captured in the national database, as these details are not required for listing. Pre-listing management could influence subsequent receipt of LRT. For example, privately insured patients may have already undergone more definitive therapy before listing, thereby reducing the need for additional LRT afterward. Currently, no national dataset reliably records pre-listing LRT stratified by insurance type. However, population-level studies of incident or early-stage HCC consistently demonstrate that privately insured patients receive more therapy overall than those with public insurance, aligning with the hypothesis that greater treatment may occur prior to listing. This consideration warrants further investigation, as existing literature on this specific point remains limited.

Furthermore, our study’s primary outcome was based on the binary finding of whether patients received LRT or not; this decision was due to inconclusive data on the efficacy of types of LRT. However, a 2021 study has shown that receipt of radioembolization or ablation as the first LRT was associated with a reduced risk of waitlist dropout, compared to other forms of LRT [7]. Given this, we hypothesized that patients with public insurance were less likely to have received these LRT options compared to patients with private insurance. However, our data showed that patients with public insurance (Medicaid: 35.72%) were just as likely, if not more, to receive such therapies, compared to patients with private insurance (33.40%) (Table 7).

**Table 7:** Procedure likelihood (ablation and Y-90 radioembolization) in relation to insurance status

	**Type of LRT received**		
Insurance	Received ablation or Y-90 radioembolization	Never received ablation or Y-90 radioembolization	Total
Private	17,526 (66.6%)	8,791 (33.4%)	26,317
Medicaid	4,401 (64.4%)	2,446 (35.7%)	6,847
Medicare	7,987 (59.3%)	5,488 (40.7%)	13,475
VA	908 (57.6%)	669 (42.4%)	1,577
Other	843 (71.0%)	345 (29.0%)	1,188

Abbreviations: VA = Veterans Affairs

The unique contribution and strength of our study lie in the exploration of the association between LRT access and insurance status, with secondary factors such as tumor burden and initial disease severity being considered and nullified as primary drivers of waitlist disparities. The novel focus on the association between LRT access and insurance status is a critical but underexplored factor in the broader context of health disparities in patients with HCC awaiting LT. This gap in the literature signifies the importance of this study. By focusing on this distinct component, we provide new insights into disparities in care delivery. Further, by means of our analysis with a national study, severity of illness, tumor burden, and LRT utilization are factors that can be excluded from association with waitlist disparities; these are important findings that deserve further exploration. Moreover, our analysis benefitted from a large sample size, encompassing 49,404 patients, which enhances the statistical power and reliability of our findings.

Our study has limitations to be considered. First, the retrospective design inherently limits the ability to establish causality and can introduce potential biases related to data collection. We evaluated LRT as a binary outcome and grouped all modalities together, without accounting for timing from HCC diagnosis to LRT initiation. Although we incorporated waitlist duration by insurance status- a factor relevant to LRT receipt- missing procedure dates precluded modeling time to treatment. While these limitations may influence long-term outcomes, they are unlikely to account for our findings, which focus on short- to intermediate-term waitlist outcomes. Treatment response could not be fully assessed using OPTN/UNOS data as defining success of treatment would require patient-level data beyond what is collected in OPTN/UNOS data. Nonetheless, this database remains a reliable resource, having been widely used in prior and current research and in informing allocation policy [8]. Lastly, because we only focus on those who are waitlisted, we are inherently comparing those patients with Medicaid that have overcome known hurdles to receiving specialized care.

In conclusion, we found Medicaid beneficiaries with HCC waitlisted for a liver transplant were not less likely to receive LRT compared to their counterparts with private insurance- a surprising finding that merits recognition and further exploration. Furthermore, baseline disease severity and tumor burden were not significantly different among patients with different insurance types, excluding their association with waitlist disparities. This understanding should shift our focus toward identifying broader structural and systemic barriers that influence waitlist outcomes of patients with HCC who are accessing care. Addressing these multifaceted variables is important to improve healthcare outcomes and ensure equitable access to liver transplantation for all patients. Further research is warranted to explore and mitigate the additional factors driving these disparities, with an objective to enhance survival rates and treatment efficacy.

## Supplementary Information

Below is the link to the electronic supplementary material.

Supplementary file1 (DTA 293915 kb)

## Data Availability

Data is provided within the manuscript or supplementary information files.
